# Oxygen-Controlled Assembly of Chiral [7]-Helicene on ZnO Surfaces

**DOI:** 10.1021/acs.langmuir.6c02922

**Published:** 2026-08-04

**Authors:** Naser Hakimi Raad, Oleksiy Lyutakov, Pin-Qian Yang, Hua Shu Hsu, Bohuslav Rezek, Egor Ukraintsev

**Affiliations:** † Faculty of Electrical Engineering, 48220 Czech Technical University in Prague, Technická 2, Prague 6 166 27, Czechia; ‡ University of Chemistry and Technology, Technická 5, Prague 6 166 28, Czechia; § Department of Applied Physics, 684788 National Pingtung University, 4-18, Minsheng Road, Pingtung 90044, Taiwan

## Abstract

Chiral molecular organization at semiconductor interfaces is vital for advancing quantum and chirality-induced spin-selective (CISS) technologies yet achieving atomic-level control remains challenging. Here, we show that the adsorption and nanoscale assembly of enantiopure [7]-helicene on Zinc Oxide (ZnO) is dictated primarily by the identity of the topmost surface atom rather than by macroscopic surface polarity. Using atomic force microscopy (AFM), circular dichroism (CD), force field molecular dynamics (FFMD), and density functional tight-binding (DFTB), we examine four ZnO terminations and find that polar O-terminated (0001) and both nonpolar surfaces reproducibly host uniform helicene nanoislands with a thickness of about 4 nm, while the polar Zn-terminated (0001) facet largely suppresses adsorption experimentally. FFMD and DFTB analyses provide a picture of the underlying interaction landscape, offering valuable theoretical support for the experimentally observed facet selectivity. The robust formation of oxygen-driven nanoislands identifies surface oxygen as an atomic-scale selector for chiral self-assembly, enabling deterministic patterning of ultrathin 2D chiral domains on oxide semiconductors. These insights establish a design rule for integrating helicene-based chiral architecture into spintronic and quantum devices.

## Graphical abstract

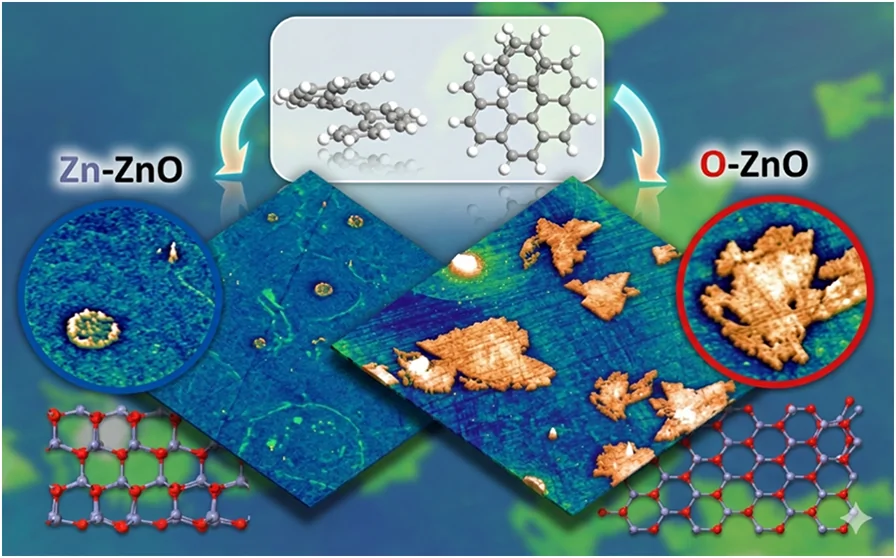

## 1 Introduction

Spin-polarized,^1,2^ optically active,^3^ and chiral^4–6^ materials are central to emerging quantum and spintronic technologies,^7,8^ where the ability to control molecular organization at solid interfaces plays a decisive role in device performance. Consequently, the development of spin-polarized,^2^ optically active,^9^ and chiral^10,11^ nanostructures is attracting growing attention in nanotechnology because they can couple structural handedness to electronic behavior. Chiral nanostructures can be produced through approaches such as hydrothermal microwave-assisted synthesis of carbon nanoparticles,^12,13^ or by functionalizing spherical gold nanoparticles with chiral organic molecules, such as [6]-helicene, which imparts chirality at Plasmon-resonant wavelengths.^13,14^ A particularly important application of chiral nanomaterials arises from the chirality-induced spin-selective (CISS) phenomenon,^15^ in which electron transmission becomes spin-dependent without requiring magnetic materials.^16,17^ This capability has motivated extensive efforts to engineer hybrid organic–inorganic interfaces that combine semiconductor functionality with molecular chirality.^17,18^

ZnO is a versatile wide-bandgap semiconductor whose properties can be tuned through doping, morphology, and facet engineering.^19–22^ Advances in controlled synthesis have produced ZnO nanostructures with well-defined crystallographic orientations, including nanocombs, nanorings, nanohelices, nanobelts, nanowires, and nanocages, each exhibiting distinct electronic and optical characteristics.^23^ These facet-dependent behaviors arise from variations in surface dipoles, atomic coordination, and defect distributions, making ZnO an ideal platform for studying how surface chemistry influences molecular adsorption and assembly. In addition, the physical and chemical properties of ZnO can be tailored through doping strategies;^24–26^ transition-metal-doped ZnO attracting considerable attention for spintronic applications.^27–30^ Other notable developments include emissive ZnO-graphene quantum dots for white-light-emitting diodes^31^ and colloidal ZnO diluted magnetic semiconductor quantum dots synthesized via the hydrolysis and condensation of zinc acetate.^32^

Beyond their role in electronic technologies, ZnO nanomaterials are increasingly employed in optically active devices and sensing applications. Chiral nanostructures of ZnO films have been shown to exhibit strong optical activity at 380 nm,^33^ while large-area 3D chiral plasmonic architectures derived from originally chiral ZnO nanopillars demonstrate pronounced optical activity throughout the visible spectrum, with promising potential for sensing, photonic, and catalytic uses.^34^ In addition, hybrid systems, such as ZnO nanoparticles functionalized with chiral porphyrin derivatives, exhibit strong chiroptical responses and stereoselective behavior, highlighting their effectiveness as chiral-selective surfaces.^35,36^ Furthermore, quantized ZnO nanocrystals have been utilized as fluorescent sensors for the chiral discrimination of amino acids, including D- and L-tryptophan.^37^

Helicenes represent a well-established class of rigid, π-conjugated chiral molecules with strong optical activity and robust structural stability.^38–41^ Their helical geometry makes them promising candidates for constructing ordered chiral layers on semiconductor surfaces.^40–43^ Prior studies have shown that helicenes can absorb on ferromagnetic metallic substrates such as Co and Fe while retaining their molecular integrity and adopting well-defined orientations.^44^ Helicene derivatives have also been explored in organic light-emitting diodes,^45,46^ surface-enhanced Raman scattering (SERS) platforms,^13,47^ and chiral spintronic systems, where certain heterohelicenes have demonstrated measurable CISS responses.^48,49^

Despite these advances, achieving atomic-level control over helicene assembly on semiconductor surfaces remains challenging.^50,51^ Molecular adsorption on ZnO is known to be highly facet-specific,^52–56^ yet the role of the terminating atomic species (Zn versus O) has not been systematically explored for chiral molecules. Understanding how surface chemistry and orientation govern helicene adsorption, is essential for designing hybrid interfaces that could eventually support spin-selective transport or chiral templating.

In this work, we investigate the adsorption and self-assembly of enantiopure P- and M-[7]-helicene on four low-index ZnO surfaces: polar Zn-terminated (0001), polar O-terminated (0001̅), and nonpolar (101̅0) and (112̅0) facets. By combining atomic force microscopy (AFM), circular dichroism (CD), force-field molecular dynamics (FFMD), and density functional tight-binding (DFTB) calculations, we examine how surface chemistry, and atomic termination influence both single-molecule interactions and collective nanoscale assembly. We find that polar O-terminated (0001) and O-containing nonpolar surfaces reproducibly give rise to 4 nm thin helicene nanoislands, whereas the polar Zn-terminated (0001) surface suppresses adsorption. This facet-dependent behavior reveals that the identity of the topmost atomic layer (rather than macroscopic polarity) is the decisive factor governing chiral assembly on ZnO.

These results establish oxygen-controlled self-assembly as a robust route for creating ultrathin 2D chiral layers on semiconductor oxides. Although spin-resolved DFTB indicates no induced magnetic moment in these systems, the ability to deterministically design chiral nanoislands provides well-defined background and guidelines for future hybrid architectures where spin-selective behavior may be introduced through magnetic doping, helicene derivatives with stronger spin-orbit coupling (SOC), or engineered transport geometries.

## 2 Results and Discussion

### 2.1 Computational Modeling of Helicene Adsorption

#### 2.1.1 Force Field Molecular Dynamics Predictions

The adsorption behaviors of a single P-[7]-helicene molecule on four low-index ZnO facets are depicted in Figures 1, 2, 3, and 4. Simulations with M-[7]-helicene were not performed, as no significant differences were expected relative to P-[7]-helicene. The computational results were obtained using two interaction models: ReaxFF (Figures 1 and 2) and Torch (Figures 3 and 4). ReaxFF was chosen for its well-established parametrization for ZnO and organic adsorbates, while Torch was incorporated to provide an independent comparison using a data-driven machine-learning potential. The results, which were done by the ReaxFF potential, show that the molecule preferentially chemically adsorbs on the polar Zn-terminated (0001) surface and both nonpolar (101̅0) and (112̅0) ZnO faces with its horizontal orientation (C atom of helicene), while it tends to remain unbound within the simulation cell on polar O-terminated (0001̅) ZnO surface (Figure 1). The vertical orientation (H atom of helicene) on polar O-terminated (0001̅) and nonpolar (101̅0) surfaces does not depict any adsorption, but polar Zn-terminated (0001) and nonpolar (112̅0) surfaces show physically weak adsorption (Figure 2).

**Figure 1:**
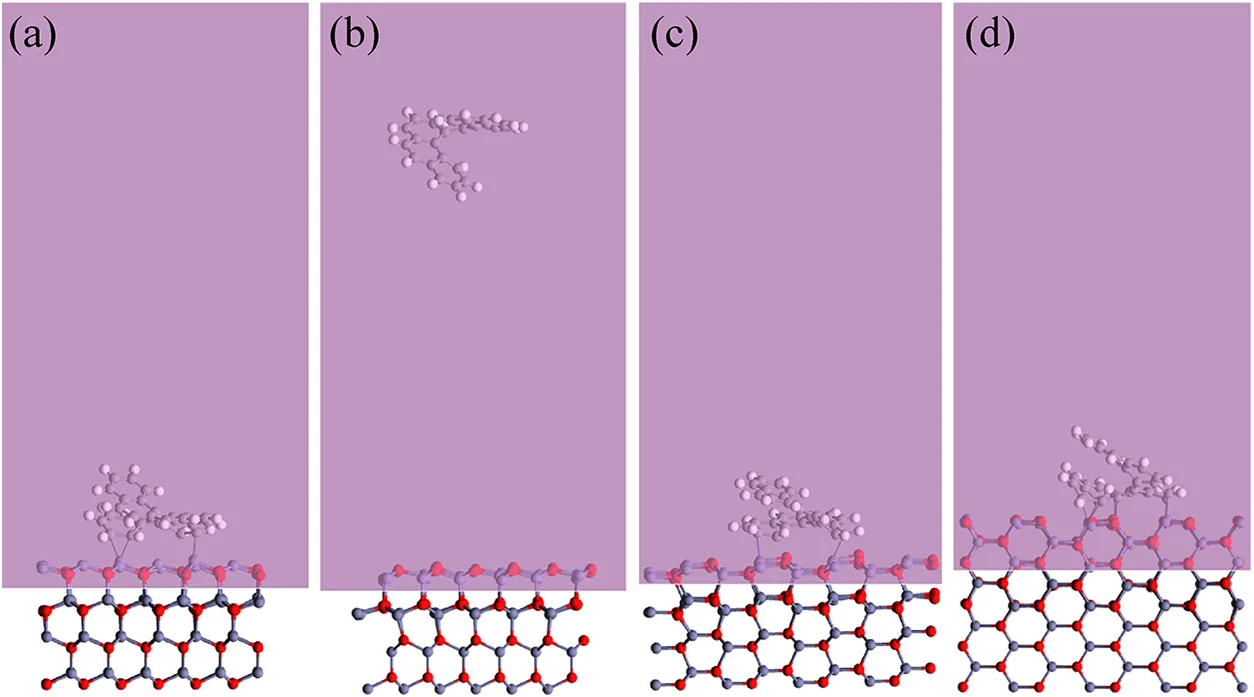
The ReaxFF-optimized structures for horizontally oriented P-[7]-helicene on four ZnO facets: (a) polar Zn-terminated (0001), (b) polar O-terminated (0001̅), (c) nonpolar (101̅0), and (d) nonpolar (112̅0).

**Figure 2:**
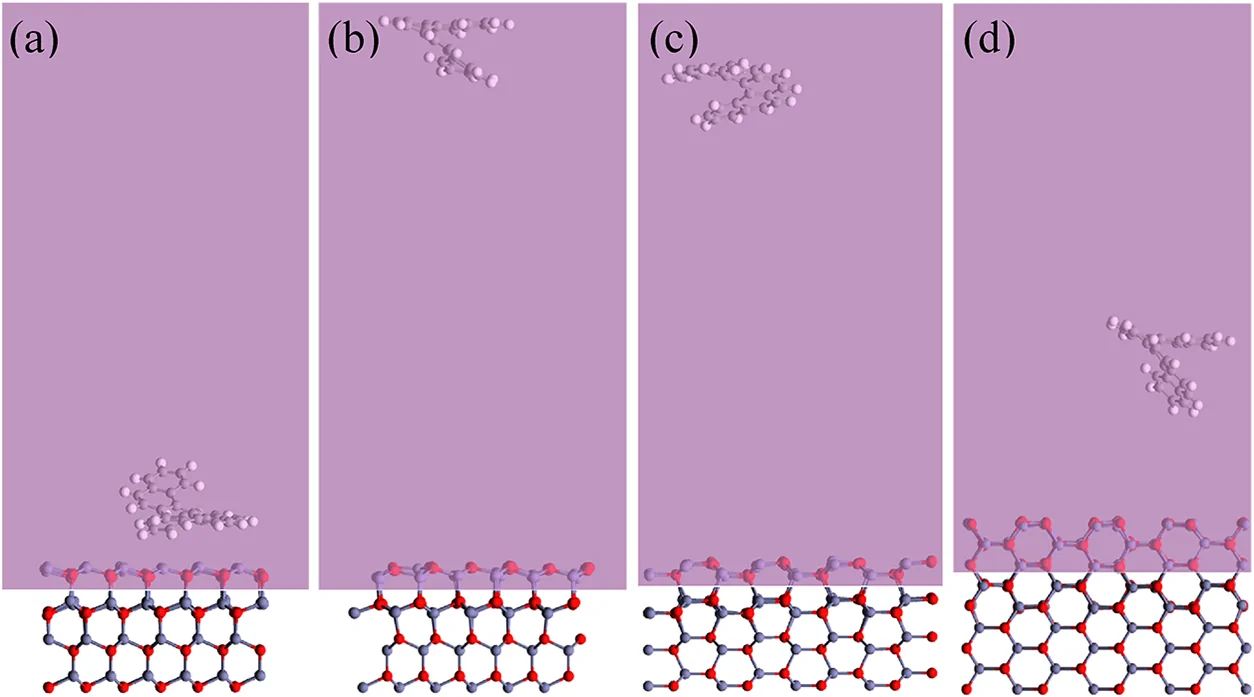
The ReaxFF-optimized structures for vertically oriented P-[7]-helicene on four ZnO facets: (a) polar Zn-terminated (0001), (b) polar O-terminated (0001̅), (c) nonpolar (101̅0), and (d) nonpolar (112̅0).

**Figure 3:**
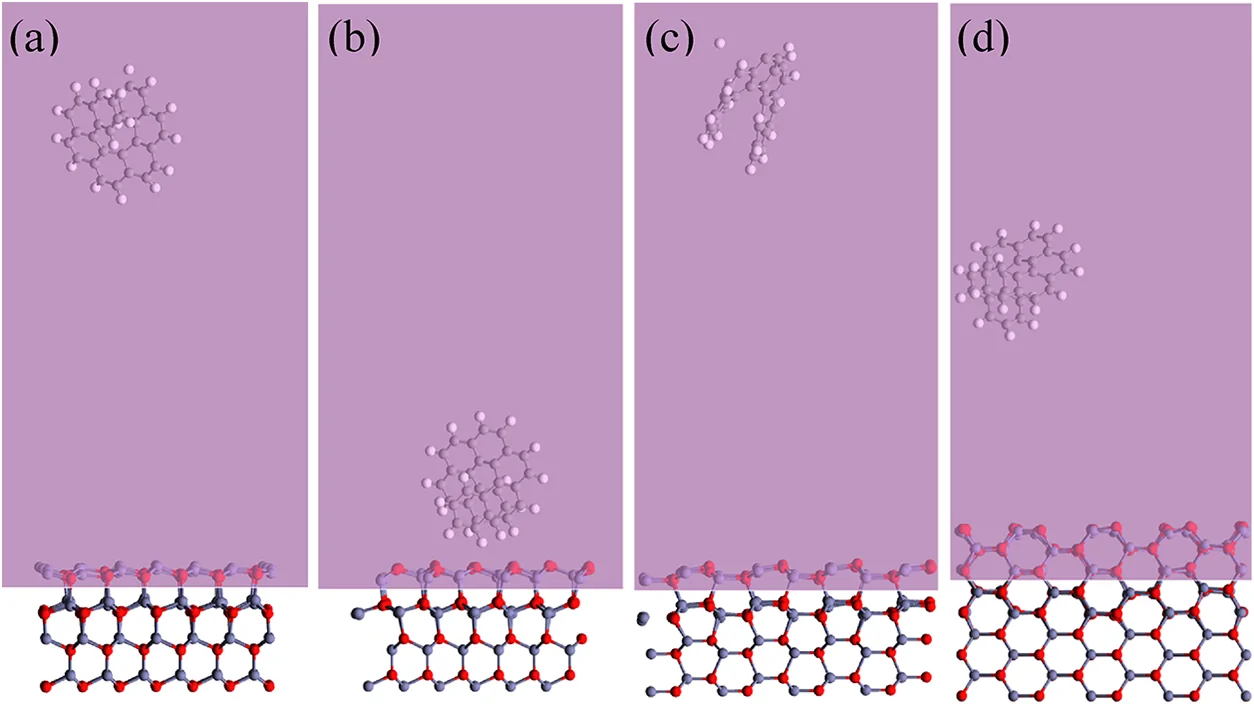
The Torch-optimized structures for horizontally oriented P-[7]-helicene on four ZnO facets: (a) polar Zn-terminated (0001), (b) polar O-terminated (0001̅), (c) nonpolar (101̅0), and (d) nonpolar (112̅0).

**Figure 4:**
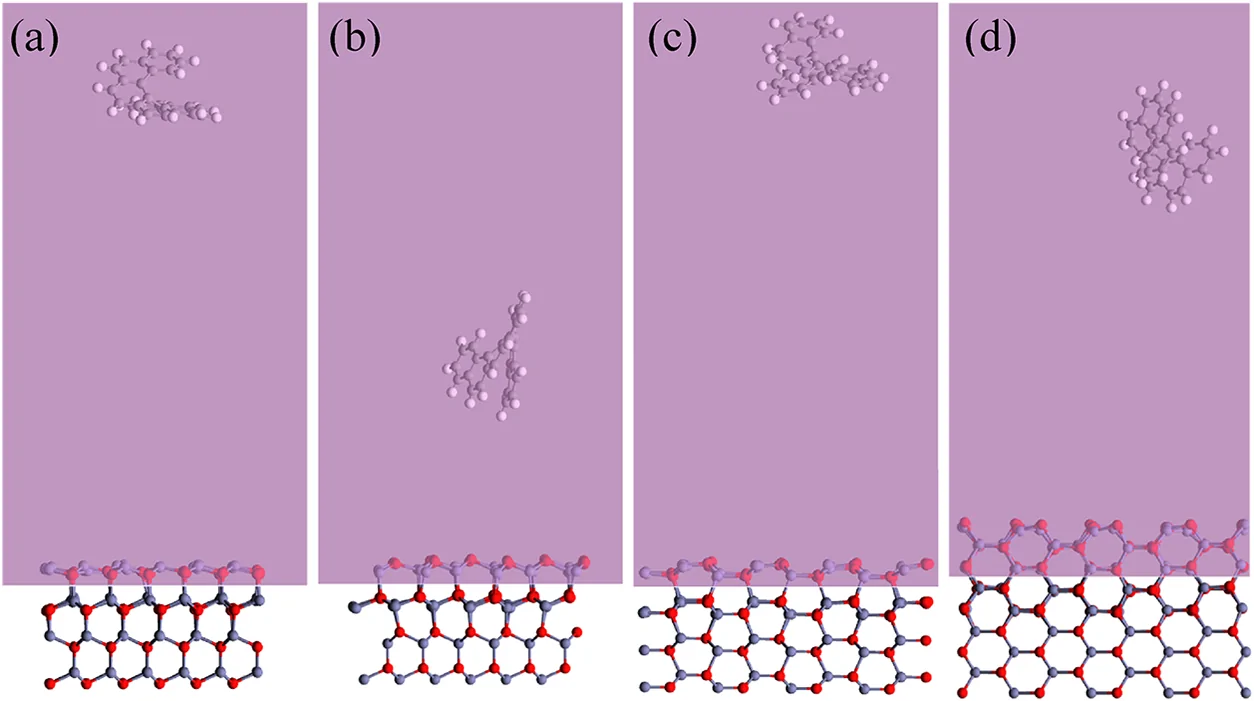
The Torch-optimized structures for vertically oriented P-[7]-helicene on four ZnO facets: (a) polar Zn-terminated (0001), (b) polar O-terminated (0001̅), (c) nonpolar (101̅0), and (d) nonpolar (112̅0).

The results of simulation with the Torch potential are demonstrated in Figures 3 and 4 for horizontal and vertical orientations of helicene molecules, respectively. The results obtained with the Torch potential follow a reverse trend compared to those of which obtained with the ReaxFF potential. In all cases, the helicene molecule was repelled except for polar O-terminated (0001̅) ZnO slab, showing weak adsorption.

ReaxFF contains parameters for π–cation interactions, because these arise naturally from its charge equilibration + dispersion + short-range attraction terms. This is why ReaxFF predicts strong adsorption on Zn-terminated surfaces, where π-cation interactions dominate. Torch as a machine-learning (ML) potential reproduce what they have seen in training. They do not automatically learn π-cation interactions unless the training set explicitly contains them. Also, ReaxFF has a mature ZnO parameter set used in catalysis, adsorption, surface chemistry, and oxide-organic interfaces. The Torch training set is unknown and likely not optimized for ZnO surfaces interacting with aromatic π-systems. This is why Torch matches AFM only on polar O-terminated surfaces (where interactions are weak and simple) but fails on Zn-terminated surfaces (where interactions are complex and highly anisotropic).

To quantify this difference, we computed pair-interaction energy curves *V*(*r*) for the O–C and Zn–C atom pairs using both force fields, scanning interatomic distances from 1.00 to 5.00 Å in 0.01 Å steps (Figure 5). Both force fields reproduce covalent minima at physically correct distances: O–C minima at 1.09 Å (ReaxFF) and 1.14 Å (Torch), close to the C≡O triple-bond length (1.13 Å); Zn–C minima at 1.96 Å (ReaxFF) and 1.99 Å (Torch), consistent with the Cambridge Structural Database average for organozinc compounds (2.01 Å). This confirms that both force fields are physically reasonable in the covalent regime.

**Figure 5:**
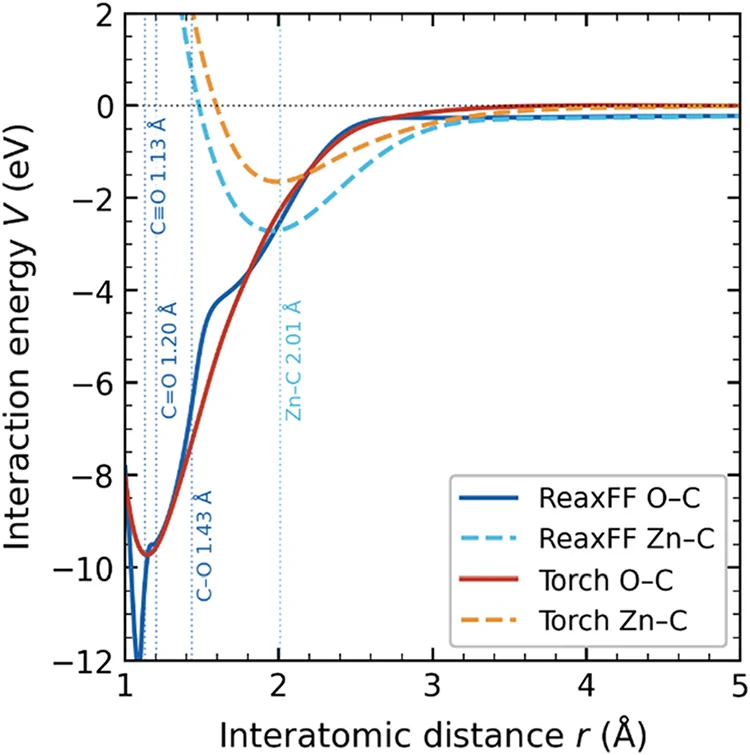
Pair-potential energy curves to explain the divergence between ReaxFF and Torch potentials.

The critical difference emerges in the van der Waals regime (*r* > 2.5 Å), which governs physisorption of helicene on ZnO. The ReaxFF potentials for both pairs plateau at approximately −220 to −290 meV and remain essentially constant between 3 and 5 Å, never approaching zero within the physically relevant interaction range. In contrast, the Torch potentials decay rapidly: at 5 Å, the Torch O–C interaction is −0.7 meV and the Torch Zn–C interaction is −8.9 meV; effectively zero for adsorption purposes. This nondecaying tail in ReaxFF introduces a persistent, distance-independent attractive offset that accumulates over the many O–C and Zn–C contacts present in a helicene/ZnO interface. On polar facets, where the balance between Zn-face and O-face interactions determines the predicted adsorption trend, this systematic offset can alter the relative stability of adsorption geometries and thereby reverse the predicted trend. Torch, with its physically decaying tail, is more sensitive to the local surface chemistry and predicts stronger face-selectivity consistent with the experimental AFM results.

In addition, the ReaxFF O–C curve exhibits a nonphysical secondary minimum near *r* = 3.1 Å (−264 meV), absent from the Torch curve. This artifact of the ReaxFF parametrization may further bias adsorption energies on the O-terminated surface, where O–C contacts are most numerous.

#### 2.1.2 DFTB Validation and Electronic Structure Analysis

The validation of these FFMD calculations was carried out using spin-polarized DFTB simulations. Helicene molecules were approached to different ZnO surfaces both vertically (H orientation) and horizontally (C orientation) with an initial distance of 3.5 Å. The vertically adsorbed helicene was not reactive enough due to positive adsorption energies for polar O-terminated (0001̅) and nonpolar (101̅0) surfaces and weak adsorption of polar Zn-terminated (0001) and nonpolar (112̅0) surfaces, while the adsorption energies for horizontally adsorbed helicene were negative, consistently with ReaxFF predictions. The findings revealed that helicene prefers to be adsorbed on Zn atom due to stronger noncovalent interactions with the cation sites and potential π-cation interaction. The π-cation interaction refers to a noncovalent attraction between the electron-rich π-system of helicene and positively charged Zn cations on the surface, which stabilizes adsorption without forming a covalent bond. The π-cation interaction occurs, because the electron-rich π-helicene interacts favorably with the Zn cations which are positively charged and electron-deficient sites.

Additionally, the amount of charge transfer between helicene molecule and ZnO surfaces was calculated using MullikenPop analysis. The results are shown in Table 1, revealing the more adsorption energy, the more charge transfer. The negative signs of Q_net_ show that the charge is transferred from helicene to the surfaces, confirming the role of Zn-centered electron-deficient sites in stabilizing π-cation interactions.

**Table 1:** Adsorption Energies and Charge Transfers of Helicene Molecule Adsorbed on Various ZnO Faces

	vertically adsorbed (H-orientation)		horizontally adsorbed (C-orientation)	
surfaces	*E*_ads_ (eV)	*Q*_net_ (e)	*E*_ads_ (eV)	*Q*_net_ (e)
polar Zn-terminated (0001)	–0.13	–0.010	–3.47	–0.72
polar O-terminated (0001̅)	+0.01	–0.018	–0.04	–0.01
nonpolar (101̅0)	+0.003	–0.016	–4.43	–0.77
nonpolar (112̅0)	–0.03	–0.028	–1.48	–0.45

For further investigation, spin-resolved projected density of states (PDOS) is presented in Figure 6. The spin-up and spin-down PDOS show the possibility whether the CISS phenomena is observed. Figure 6 shows the PDOS of both spin-up and spin-down for pure, horizontally adsorbed, and vertically adsorbed helicene molecules on: (a) polar Zn-terminated (0001); (b) polar O-terminated (0001̅); (c) nonpolar (101̅0); (d) nonpolar (112̅0) ZnO surfaces, respectively. All figures demonstrate a symmetrical shape between spin-up (red) and spin-down (blue) electrons, indicating a zero total magnetic moment and no magnetic orientation. This means that the presence of P-[7]-helicene molecule does not stimulate the ferromagnetic property into the ZnO surfaces at room temperature. From these graphs, it also can be seen that the presence of helicene molecule reduces the band gap, still retaining semiconductor behavior. These results thus show that while helicene interacts strongly with Zn-terminated surfaces, no CISS-related spin splitting is observed in the electronic structure.

**Figure 6:**
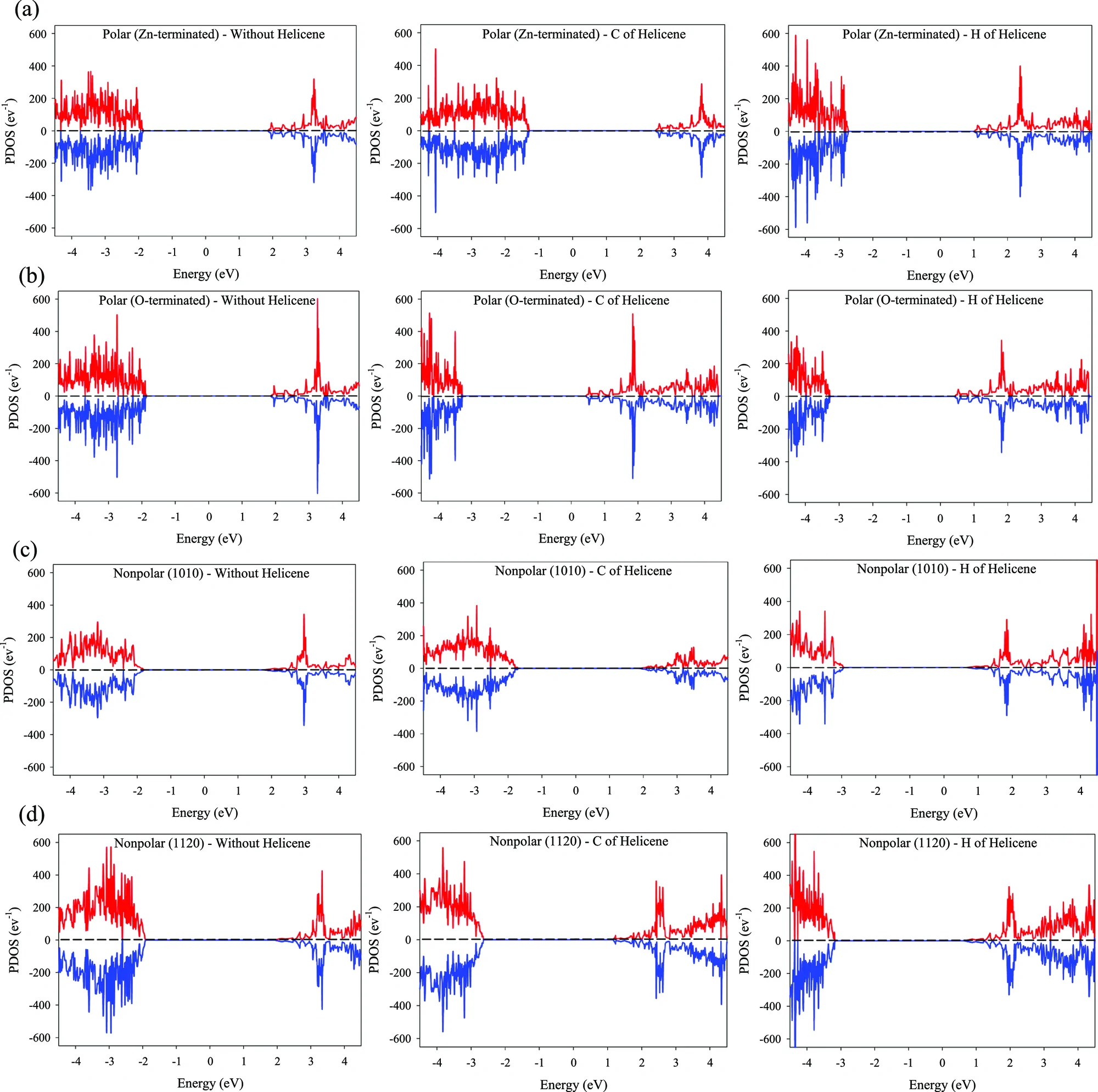
Projected density of states. (a) Pure, horizontally adsorbed, and vertically adsorbed helicene on polar Zn-terminated (0001) ZnO surfaces. (b) Pure, horizontally adsorbed, and vertically adsorbed helicene on polar O-terminated (0001̅) ZnO surfaces. (c) Pure, horizontally adsorbed, and vertically adsorbed helicene on nonpolar (101̅0) ZnO surfaces. (d) Pure, horizontally adsorbed, and vertically adsorbed helicene on nonpolar (112̅0) ZnO surfaces.

Having established theoretical predictions for helicene adsorption, we turned to CD and AFM characterization to determine whether similar facet-specific assembly occurs experimentally.

### 2.2 Structural Characterization of ZnO Substrates; X-ray Diffraction Measurements

The interaction between helicene molecules and ZnO surfaces was examined in greater detail using several experimental methods. Before depositing the helicenes, X-ray diffraction (XRD) analyses were conducted on all four ZnO surface terminations to verify their crystallographic orientation and single-crystal quality (Figure 7). For the c-plane polar Zn-terminated (0001) and polar O-terminated (0001̅) ZnO surface terminations, the XRD patterns feature a prominent (002) reflection at 34.29° along with a weaker (1012)/(104) reflection at 72.45°. The *a*-plane nonpolar (101̅0) ZnO surface displays a strong (100) peak at 31.64° and a less intense (103) peak at 66.26°, whereas the m-plane nonpolar (112̅0) surface was characterized by a dominant (110) reflection at 56.47°. These diffraction results align with previous reports^57^ and confirm that all ZnO samples studied are monocrystalline.^58^

**Figure 7:**
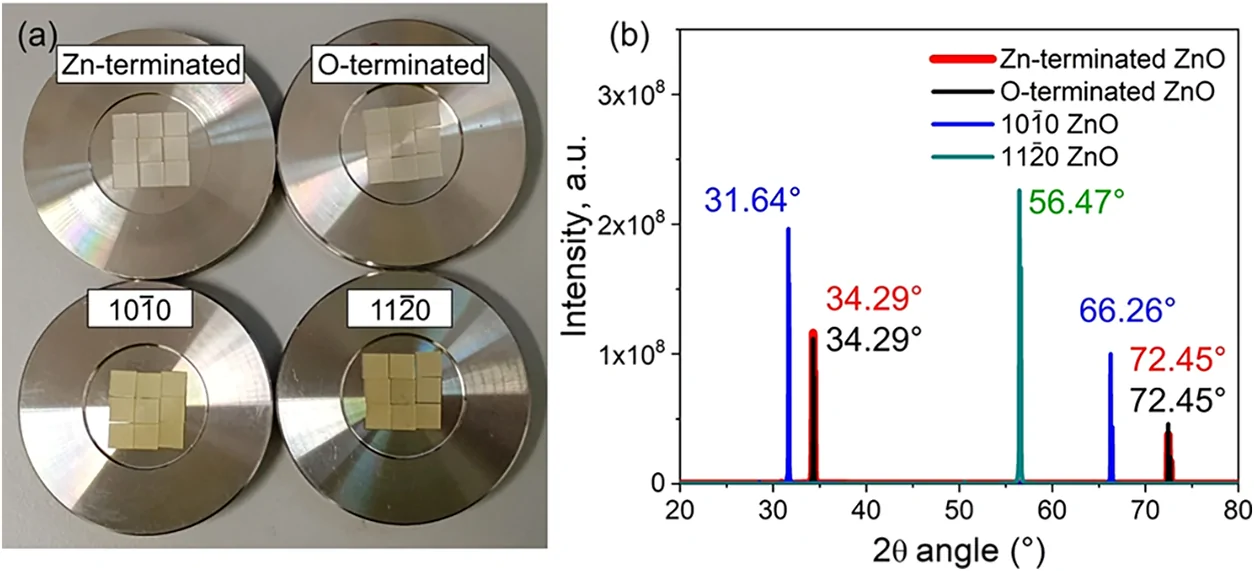
(a) Optical images of ZnO samples. (b) XRD spectra of four ZnO surface terminations before helicene deposition.

### 2.3 Optimization of Helicene Deposition Protocols

Based on our earlier studies,^54,56^ this work concentrates on the deposition of monolayer or submonolayer coverages of helicene molecules, deliberately avoiding the formation of thick molecular layers. Single-molecule deposition^44^ is also excluded, since our objective is to examine the self-assembly behavior and layer formation of helicene on low-index ZnO surfaces.

To determine suitable helicene concentrations and deposition conditions, we conducted preliminary adsorption experiments on polar Zn-terminated (0001) and nonpolar (101̅0) ZnO samples.

Initially, a P-[7]-helicene solution (*c* = 10^–3^ g/L in dichloromethane, DCM) was drop-casted on polar Zn-terminated (0001) ZnO and allowed to dry, which led to the formation of nonuniform, salt-like fractal structures. Lowering the concentration to 10^–6^ g/L and applying drop-casting to nonpolar (101̅0) ZnO yielded approximately 4.4 nm-thick nanolayered structures; however, the surface coverage was still highly uneven, characterized by large aggregates in the central region and almost bare areas near the edges. The rapid evaporation of DCM resulted in locally varying concentrations, prompting us to discard drop-casting as an effective deposition approach.

The (101̅0) nonpolar ZnO sample was placed in a glass tube containing P-[7]-helicene solution (*c* = 10^–6^ g/L in DCM) for 4 days, after which it was rinsed with DCM and dried under a nitrogen flow. This procedure yielded nanoislands approximately 4 nm-thick with a much more uniform spatial distribution of helicene molecules, consistent with observations from previous studies.^54,56^

Consequently, this deposition protocol was used for all ZnO samples using a helicene concentration of 10^–6^ g/L in DCM.

### 2.4 Morphology of Adsorbed Helicene Layers studied by Atomic Force Microscopy

Figure 8 shows the resulting helicene nanostructures formed by adsorption on different ZnO surface facets. The ZnO surface facets display typical polishing-related features in the background, noticeable on nonpolar (101̅0) ZnO sample (Figure 8c). After the P-[7]- and M-[7]-helicene deposition, the polar Zn-terminated (0001) surface remained largely free of adsorbates, with only sporadic aggregates observed (Figure 8a,e). By contrast, nanoislands with a typical thickness of about 4 nm and the lateral size of 1 μm are developed on the polar O-terminated (0001̅) as well as on both nonpolar (101̅0) and (112̅0) surfaces (Figure 8b–d,f–h). In many cases, these nanoislands frequently displayed a higher central feature. For each sample, six independent 10 × 10 μm^2^ AFM scans were acquired at different locations on the surface, and the most representative image was selected for presentation in Figure 8. No statistically significant differences in nanoislands density, height, or morphology were observed between P-[7]- and M-[7]-helicene on any of the four ZnO facets, within the resolution of our AFM measurements. This is consistent with the qualitative similarity already visible between Figures 8(a–d),(e–h). Although we cannot fully exclude trace chemical impurities below these detection limits, the consistency of nanoislands formation across multiple independent depositions on each facet type argues against a dominant role of irreproducible contamination.

**Figure 8:**
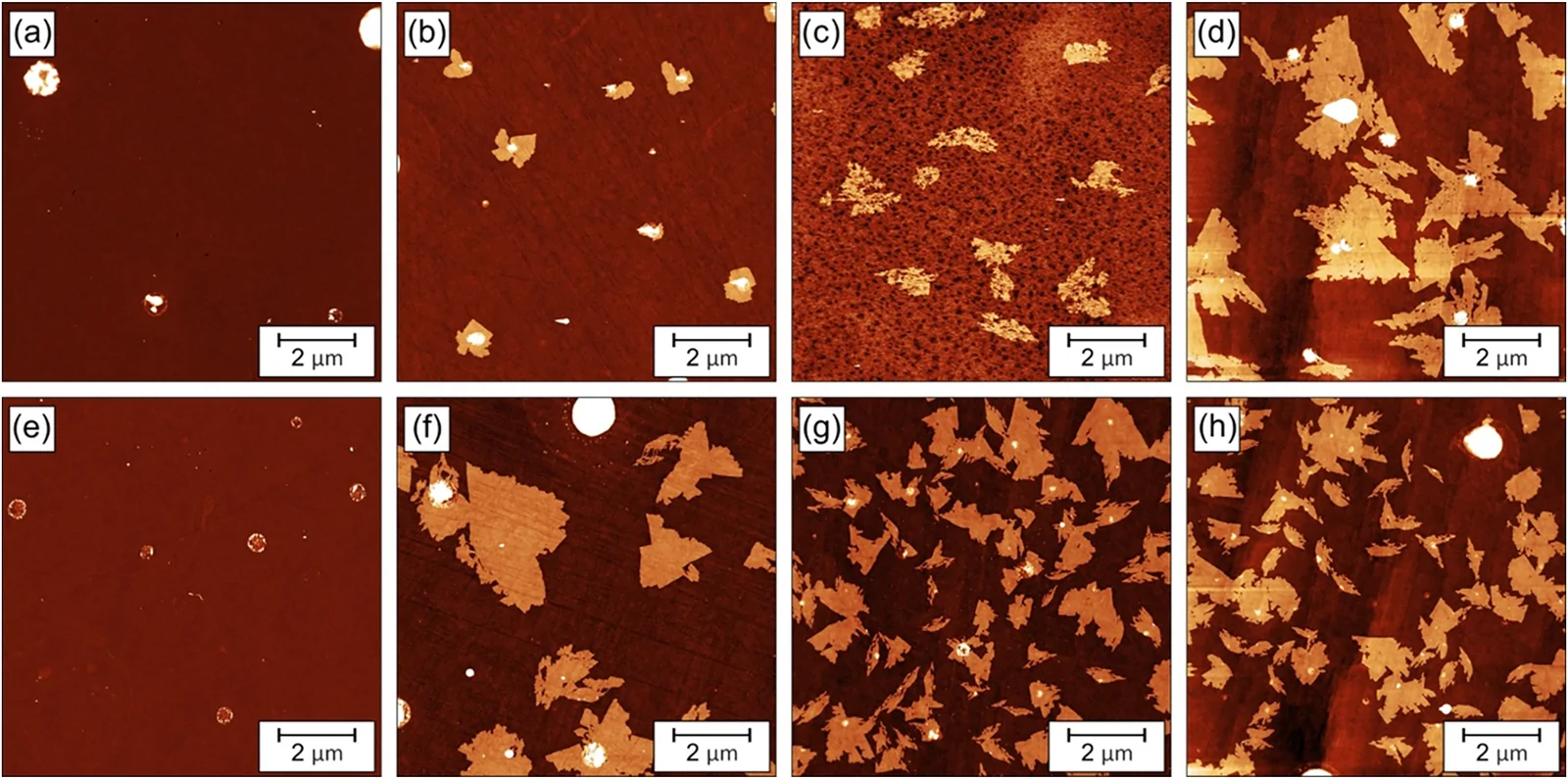
Result of P-[7]-helicene (a–d) and M-[7]-helicene (e-h) adsorption on (a, e) polar Zn-terminated (0001); (b, f) polar O-terminated (0001̅); (c, g) nonpolar (101̅0); (d, h) nonpolar (112̅0) ZnO samples studied by AFM. Image size is 10 × 10 μm^2^. Z-scale is 10 nm.

The adsorption of helicene on low-index ZnO surfaces is similar to the behavior previously reported for thiorphan molecules,^56^ with the primary distinction occurring on the polar O-terminated (0001̅) surface. There, helicene assembles into nanoislands that sometimes exhibit a central elevation, while thiorphan more commonly forms spherical aggregates.

We can explain the discrepancy between experimental and theoretical results in Figures 1 and 2 namely that helicene does not adsorb on the polar O-terminated (0001̅) ZnO surface, by the fact that in the experiment we only have the polar O-terminated (0001̅) ZnO surface and a few cm of DCM above it. In contrast, in the FF calculations, due to periodic boundary conditions, we also have the polar Zn-terminated (0001) ZnO surface, 5 nm above the polar O-terminated (0001̅) ZnO surface, to which the attraction is an order of magnitude stronger than the polar O-terminated (0001̅) ZnO surface, so the helicene molecules move upward (toward the polar Zn-terminated (0001) ZnO surface). On the other hand, Figure 3 obtained with the Torch potential clearly indicates adsorption of helicene molecule to polar O-terminated (0001̅) ZnO surface.

These findings reveal that the ReaxFF potential is generally more consistent with DFTB simulations and AFM measurements except polar surfaces, the Torch potential consistently describes the AFM results on polar surfaces.

### 2.5 Chiroptical Properties at Sub-Monolayer Coverage; CD measurements

Figure 9 presents the CD and absorbance spectra for P-[7]-helicene and M-[7]-helicene deposited on four different ZnO surfaces. The black arrows at 270 and 350 nm indicate the characteristic CD features of P-/M-[7]-helicene in DCM, while the red arrow at approximately 400 nm marks the ZnO absorption edge. In the present samples, no distinct CD signatures attributable to helicene are observed within the measured spectral range. Instead, all prominent spectral features coincide with ZnO-related optical transitions.

**Figure 9:**
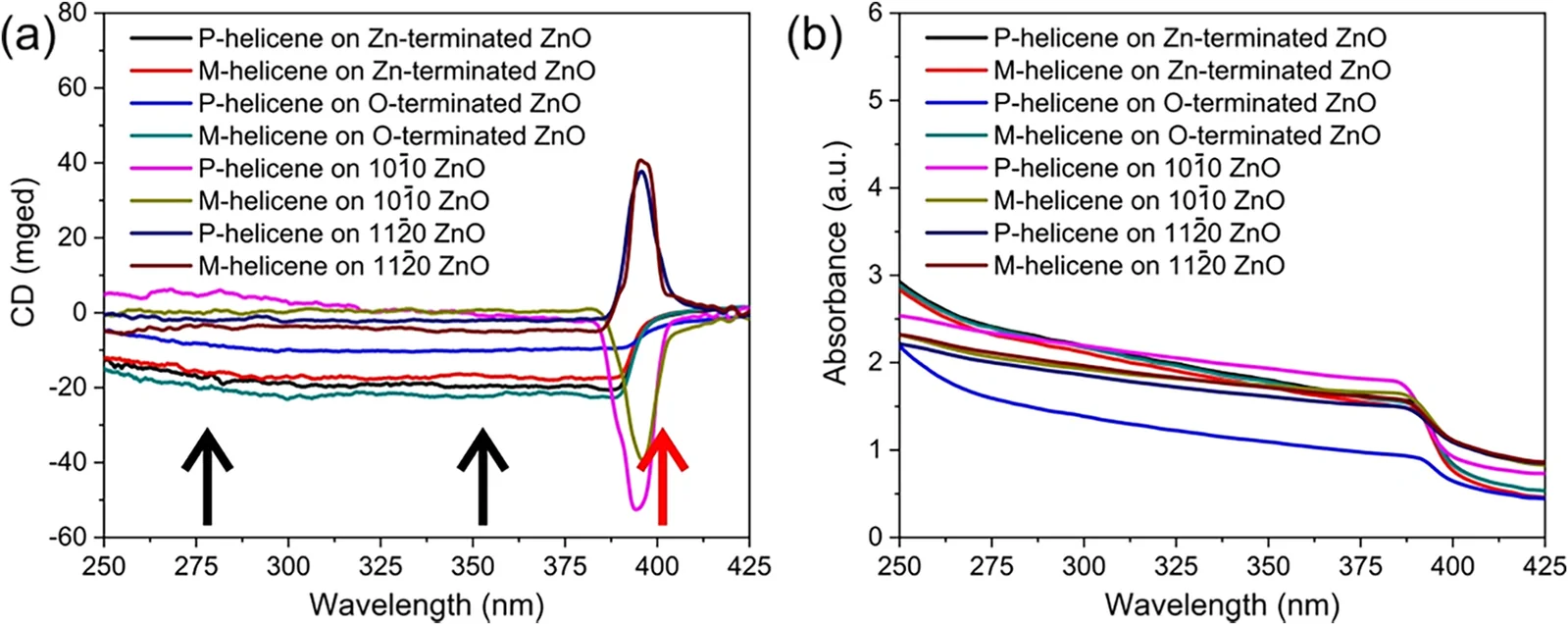
(a) The CD results of P-[7]-helicene and M-[7]-helicene adsorption on four ZnO surfaces. Black arrows at 270 and 350 nm correspond to positions of expected signal from helicene molecules. Red arrow at 400 nm corresponds to the position of expected signal from ZnO. (b) The absorbance results of P-[7]-helicene and M-[7]-helicene adsorption on four ZnO surfaces.

This indicates that at submonolayer coverage, the optical response is dominated by the ZnO substrates, while the helicene layer does not provide a measurable contribution to the CD signal within the sensitivity of our setup. Consequently, the observed CD spectra primarily reflect intrinsic ZnO electronic transitions rather than molecular chiroptical activity.

The absence of detectable molecular CD is consistent with known surface-induced suppression of chiroptical activity: adsorption constrains molecular orientation, reducing the projection of transition dipoles along the optical axis. Differences in CD line shape across facets arise from intrinsic variations in ZnO surface electric fields, band bending, and defect states.

Thus, the negligible CD signal does not contradict AFM evidence of ordered chiral assembly; rather, it reflects an established phenomenon in surface-adsorbed chiral systems.

### 2.6 Comparative Analysis and Interpretation

AFM and FF/DFTB observations raise important questions about the role of surface chemistry, surface polarity and simulation accuracy, which we address below.

Surface chemistry and surface polarity are key factors that typically determine both the interaction strength and the resulting organization of adsorbed molecules. Variations in chemistry or polarity generate distinct electrostatic environments across different ZnO facets, directly influencing whether helicene molecules assemble into ordered nanoislands or remain sparsely distributed. Consequently, these parameters play a critical role in controlling chiral self-assembly on semiconductor surfaces. AFM measurements indicate that the presence of oxygen atoms in the topmost layer promotes the formation of nanoislands, whereas DFTB and ReaxFF predict stronger adsorption on Zn-terminated surfaces due to π-cation interactions. This mismatch highlights limitations in theoretical models: ReaxFF accurately captures dispersion-dominated adsorption on nonpolar surfaces but overestimates π-cation interactions on polar Zn-terminated surfaces, Torch reproduces weak interactions on O-terminated surfaces but fails to capture π-cation effects entirely, and periodic slab boundary conditions introduce artificial attractions, not present in solution-phase experiments.

Despite these differences, all approaches converge on a key insight: surface termination, not macroscopic polarity, governs chiral assembly on ZnO.

The adsorption of chiral helicene molecules on low-index ZnO surfaces, along with the emergence of approximately 4 nm-thick nanoislands composed of chiral monomers, is particularly noteworthy. This behavior offers a potential route for the controlled fabrication of well-defined nanoislands from chiral molecules. Numerous studies have emphasized the critical role of molecular chirality in adsorption phenomena.^59,60^ Comparable nanoislands of around 4 nm thickness, as well as multilayer assemblies, have also been reported for another small molecule, thiorphan on ZnO.^57^ It is therefore reasonable to expect similar for the other molecular systems on different surfaces; for instance, helicene-based macro-cycles have been reported to form nanoislands on HOPG.^60^ The presence of in-plane dipole moments on nonpolar (101̅0) and (112̅0) ZnO surfaces likely directs molecular assembly, promoting the formation of nanoislands with a well-defined thickness. Compared to our previous studies on ZnO^52–54^ in which AFM data were consistently explained by simulations, helicene adsorption on low-index ZnO facets exhibits markedly different behavior in DFTB, FFMD and AFM studies. This discrepancy suggests that some relevant interactions may not be fully captured by the theoretical simulations.

One potential application of this study is the functionalization of ZnO nanostructures- such as nanoparticles, nanocombs, nanorings, and nanorods with chiral helicene molecules. The ability to form thin helicene layers on different low-index ZnO facets could facilitate the development of chiral shells surrounding these nanomaterials. Chiral core–shell nanoparticles have been demonstrated in earlier studies.^61–65^ Our findings indicate that a chiral coating on ZnO nanoparticles may be achievable. Nevertheless, direct experimental confirmation on nanoparticles has not yet been possible, primarily because suitable instrumentation is lacking to reliably verify the presence of an approximately 4 nm thick helicene layer on 20 nm ZnO nanoparticles and to characterize the resulting core–shell structures. Common techniques such as dynamic light scattering (DLS) and AFM lack the resolution required to detect such small variations, while CD measurements are likely insufficiently sensitive.

While single-atom adsorption, for example Co, was not examined in this work, earlier studies have demonstrated that the isolated [7]-helicene molecule on ferromagnetic Co and Fe thin films allow microscopic probing of the CISS effect under well-defined vacuum and atomic conditions.^44^ More recent research indicates that the chirality of organic molecules can be harnessed in the design of spintronic devices, offering a feasible pathway for implementing the CISS effect.^66^ Discrete nanoislands composed of chiral molecules on ZnO surfaces could function as localized spin carriers, creating confined regions where spin polarization can be generated and maintained. These nanoislands are especially attractive for developing spin-polarized ZnO nanostructures, since their controlled dimensions and morphology may promote spin-selective transport, while limiting spin relaxation. Helicene-based nanoislands could serve as localized chiral domains in spintronic architectures, although additional investigations involving nanoparticle systems are still required. Precise control over the assembly and orientation of such chiral nanoislands would enable the design of surfaces with tunable spin-dependent properties, paving the way for spintronic applications such as spin filters and chiral quantum information components. Integrating the inherent electronic properties of ZnO with CISS at the nanoscale, thus offers a promising route toward stable, room-temperature spintronic technologies.

These findings underscore the importance of surface chemistry in directing chiral molecular assembly, which is central to exploiting the CISS effect. Although CD measurements at submonolayer coverage were below detection limits, the formation of well-defined helicene nanoislands on both nonpolar (101̅0), (112̅0) and polar O-terminated (0001̅) ZnO surfaces suggest potential for creating localized chiral domains. Such domains could act as spin filters or spin-polarized regions in ZnO-based architectures, paving the way for hybrid spintronic devices operating at room temperature. By integrating semiconducting properties of ZnO with helicene-induced chirality, this work provides a foundation for designing surfaces with tunable spin-dependent functionalities.

## 3 Experimental Section

### 3.1 XRD Measurements

XRD measurements were carried out using an XRDynamic 500 diffractometer with Cu Kα radiation (λ = 1.5405 Å) before the deposition of helicene molecules. Data were collected from nine ZnO samples (5 × 5 mm^2^ each) arranged in a 3 × 3 array. The acquisition time was 1 h, covering a 2θ range of 15–80°, for four different low-index ZnO surface terminations.

### 3.2 Samples Preparation

ZnO substrates consist of single-crystalline plates (5 × 5 × 0.5 mm^3^) with different surface terminations; polar Zn-terminated (0001), single-side polished polar Zn-terminated (0001), polar O-terminated (0001̅), single-side polished polar O-terminated (0001̅), and nonpolar (101̅0) and (112̅0) orientations. All substrates were purchased from MSE supplies and had C-plane ⟨0001⟩ orientation within ±0.5°, 99.99% purity, and lattice parameters of *a* = 3.252 Å, *c* = 5.313 Å.

Prior to helicene adsorption, all as-received ZnO samples were rigorously cleaned: first in ethanol in an ultrasonic bath to remove residues from mechanical polishing, and then in DCM to ensure the removal of residual organic impurities, followed by drying under a nitrogen stream.

Enantiopure P- and M-[7]-helicene (synthesized and separated by I. Panov, ICPF, Prague) were dissolved in DCM to prepare a stock solution with a concentration of 10^–3^ g/L. Each ZnO sample was placed in a 15 mL glass cuvette containing 10 mL of DCM, after which 10 μL of the stock solution was added, yielding a final helicene concentration of 10^–6^ g/L. After 4 days of deposition, the ZnO samples were rinsed with DCM to remove weakly adsorbed helicene molecules and dried with nitrogen.

### 3.3 AFM Measurements

The surface morphology of adsorbed helicene molecules was examined using AFM topography measurements. These measurements were carried out on an ICON AFM system (Bruker) operating in PFQNM mode with CF_4_ plasma^67^ treated Multi75Al cantilevers (BudgetSensors). The imaging parameters were as follows; scan area of 10 × 10 μm^2^, image resolution of 1024 × 1024 px^2^, scan rate of 0.1 Hz, gain of 1.5, dz = 20 nm, Z-scale = 1 μm, and force threshold = 1 nN. All AFM data were processed and analyzed using Gwyddion software.^68^

### 3.4 Circular Dichroism Measurements

To verify the self-organized assembly of chiral helicene molecules on single-crystal ZnO surfaces mediated by the CISS effect, CD measurements were performed using a Jasco-J815 CD spectrometer. The instrument is equipped with a 450 W xenon lamp that serves as the light source and is first converted into linearly polarized light by a polarizer. A photoelastic modulator then alternates the polarization between left- and right-handed circular states at a frequency of 50 kHz. The modulated light beam passes perpendicularly through the single-crystal ZnO substrate bearing the adsorbed chiral helicene molecules, and the resulting CD signal is collected to probe the molecular organization on the ZnO surface.

### 3.5 Force Field Molecular Dynamics simulations

The geometry optimization of the P-[7]-helicene molecule adsorbed on both polar and nonpolar ZnO surfaces was carried out using FF calculations within the QuantumATK software.^69,70^ To ensure consistency with DFTB simulations and AFM observations, two different interaction models were employed, including ReaxFF (a classical reactive force field)^71^ and Torch (a machine-learning interatomic potential).^72^ An integration time-step of 50000 ps (picosecond) was employed for all simulations with NVT Nose Hoover ensemble. In the FF framework, interactions are primarily described using simple harmonic terms, with the helicene molecule modeled as a set of atoms interacting with the ZnO nanostructures through a parametrized potential that approximates the true interatomic interactions. The suitability of the ReaxFF potential for modeling interactions between ZnO surfaces and biomolecules has been demonstrated in previous studies.^73^ These FF-based calculations were used to determine the most likely orientation of the P-[7]-helicene molecule on different ZnO surface slabs. Two initial adsorption configurations were considered: a vertical arrangement (H orientation of the helicene molecule) and a horizontal arrangement (C orientation of the helicene molecule). In both cases, the molecule was placed above the ZnO surface in vacuum with an initial separation of 3.5 Å. The ZnO slabs were constructed by cleaving bulk ZnO along selected Miller index planes, yielding supercells with dimensions of 20 × 20 × 50 Å. By analyzing the optimization trajectories and total energies, the most energetically stable molecular orientation on each ZnO surface was determined. During the optimization, the two innermost layers of each slab were fixed along the *c*-axis. Solvent effects were included using an implicit DCM solvent model (ε = 8.93) to account for its influence on molecular structure, energetics, and dynamics, rather than treating the solvent as a reactive species.

### 3.6 DFTB Simulations

To corroborate and complement the FF results, the DFTB method was employed to examine the electronic and magnetic characteristics of the proposed system. DFTB is a computationally efficient quantum mechanical approach based on a second-order expansion of the Kohn–Sham total energy with respect to charge-density fluctuations.^74^ The Slater-Koster parameter set DFTB (dftb.org, znorg-0–1) was used, which offers a minimal basis and has been extensively validated for covalently bonded materials. All simulations utilized a Fermi–Dirac occupation scheme with a broadening temperature of 300 K. A density mesh cutoff of 150 Ry was chosen to ensure numerical precision. Brillouin-zone sampling was performed using a 2 × 2 × 1 Monkhorst–Pack k-point grid, with periodic boundary conditions applied in *a*-axis and *b*-axis. Structural relaxation proceeded until all atomic forces were reduced below 0.05 eV/Å. Spin-polarized Kohn–Sham equation was adopted to account for magnetic effects.

The adsorption energies (*E*_ads_) of helicene molecule on ZnO surfaces were computed using

1 $$E_{\mathrm{ads}}=E_{S}+E_{H}-E_{\left\{S+H\right\}}$$

where *E*_S_ and *E*_H_ denote the total energies of the isolated ZnO surface and the free helicene molecule, respectively, and *E*_{S+H}_ represents the total energy of the combined system when helicene is adsorbed on the surface.

Additionally, charge transfer was evaluated using MullikenPopulation analysis. The net charge transfer between helicene and ZnO slabs was determined as

2 $$Q_{\mathrm{net}}=Q_{\left\{S+H\right\}}-Q_{H}$$

where *Q*_{S+H}_ is the total charge of the ZnO slab with the helicene molecule present, and *Q*_H_ corresponds to the total charge of the isolated helicene molecule.

## 4 Conclusions

Our study shows that the assembly of enantiopure P- and M-[7]-helicene on ZnO slabs is controlled by one key factor: only surfaces with oxygen in the topmost atomic layer support the formation of chiral nanoislands. Across four low-index ZnO terminations: polar Zn-terminated (0001), polar O-terminated (0001̅), nonpolar (101̅0), nonpolar (112̅0) ZnO surfaces, we combined AFM, CD, FFMD, and DFTB analyses to build a consistent picture of how surface chemistry, rather than macroscopic polarity, and atomic species direct adsorption strength and chiral molecular organization.

AFM measurements show that the polar O-terminated (0001̅) and both nonpolar surfaces support formation of the helicene nanoislands with a thickness of approximately 4 nm, while the polar Zn-terminated (0001) surface remains largely bare for both helicene enantiomers. CD spectra confirm clean interfaces and facet-dependent ZnO optical signatures, while the absence of detectable helicene CD reflects the well-known suppression of chiroptical activity at submonolayer coverage. FFMD and DFTB simulations clarify the underlying interaction landscape, highlighting the role of π-cation interactions on Zn sites and explaining why theoretical predictions diverge from experimental behavior on polar surfaces.

Spin-resolved DFTB calculations show symmetric spin-up and spin-down densities, indicating that helicene adsorption does not induce magnetic moments or spin polarization in ZnO under the present conditions. Although no CISS signature is detected, the ability to deterministically pattern chiral nanoislands on selected ZnO facets provides a clean platform for future hybrid architectures. The results establish oxygen-controlled self-assembly as a robust route for creating ultrathin 2D chiral layers on semiconductor oxides. These chiral domains could be integrated with magnetic dopants, helicene derivatives with stronger spin–orbit coupling, or engineered transport geometries to introduce spin-selective behavior.

By revealing how atomic-scale termination governs chiral molecular organization, it thus provides essential design rules for integrating helicene-based chiral architectures into future spintronic and quantum device platforms.

## Data Availability

The data underlying this contribution are accessible on the Zenodo repository under the following DOI: 10.5281/zenodo.18324366.

## Abbreviations

AFM: atomic force microscopy
CISS: chirality-induced spin selectivity effect
DCM: dichloromethane
FFMD: force field molecular dynamics simulations
CD: circular dichroism
XRD: X-ray diffraction

## References

[1] Inomata K., Ikeda N., Tezuka N., Goto R., Sugimoto S., Wojcik M., Jedryka E. (2008). Highly Spin-Polarized Materials and
Devices for Spintronics. Sci. Technol. Adv.
Mater..

[2] Sun Y., Zhuo Z., Wu X., Yang J. (2017). Room-Temperature Ferromagnetism
in Two-Dimensional Fe_2_ Si Nanosheet with Enhanced Spin-Polarization
Ratio. Nano Lett..

[3] Li X., Wu F., Yao Y., Wu W., Ji C., Li L., Sun Z., Luo J., Liu X. (2022). Robust Spin-Dependent Anisotropy
of Circularly Polarized Light Detection from Achiral Layered Hybrid
Perovskite Ferroelectric Crystals. J. Am. Chem.
Soc..

[4] Naaman R., Waldeck D. H. (2015). Spintronics and Chirality: Spin Selectivity in Electron
Transport Through Chiral Molecules. Annu. Rev.
Phys. Chem..

[5] Long G., Sabatini R., Saidaminov M. I., Lakhwani G., Rasmita A., Liu X., Sargent E. H., Gao W. (2020). Chiral-Perovskite Optoelectronics. Nat. Rev. Mater..

[6] Ben
Dor O., Morali N., Yochelis S., Baczewski L. T., Paltiel Y. (2014). Local Light-Induced Magnetization Using Nanodots and
Chiral Molecules. Nano Lett..

[7] Zhang W., Yang T., Jiang S., Ding F., Liu Y., Wang M., Li W., Feng J., Deng M., Yang S., Zhai Y. (2025). Disordered and Conductive
Chiral Spin-Selective Strategy to Enhance Small-Molecule-Based Spintronic
Application. Small.

[8] Shang Z., Liu T., Yang Q., Cui S., Xu K., Zhang Y., Deng J., Zhai T., Wang X. (2022). Chiral-molecule-based
spintronic devices. Small.

[9] Kang L., Jenkins R. P., Werner D. H. (2019). Recent Progress in Active Optical
Metasurfaces. Adv. Opt. Mater..

[10] Wang L., Urbas A. M., Li Q. (2020). Nature-Inspired Emerging Chiral Liquid
Crystal Nanostructures: From Molecular Self-Assembly to DNA Mesophase
and Nanocolloids. Adv. Mater..

[11] Nagata Y., Takeda R., Suginome M. (2019). Asymmetric catalysis in chiral solvents:
chirality transfer with amplification of homochirality through a helical
macromolecular scaffold. ACS Cent. Sci..

[12] Đorđević L., Arcudi F., D’Urso A., Cacioppo M., Micali N., Bürgi T., Purrello R., Prato M. (2018). Design Principles of
Chiral Carbon Nanodots Help Convey Chirality from Molecular to Nanoscale
Level. Nat. Commun..

[13] Guselnikova O., Elashnikov R., Švorčík V., Záruba K., Jakubec M., Žádný J., Storch J., Lyutakov O. (2023). Charge-Transfer Complexation: A Highly
Effective Way towards Chiral Nanoparticles Endowed by Intrinsically
Chiral Helicene and Enantioselective SERS Detection. Sens. Actuators B Chem..

[14] Balogh D., Zhang Z., Cecconello A., Vavra J., Severa L., Teply F., Willner I. (2012). Helquat-induced
chiroselective aggregation
of Au NPs. Nano Lett..

[15] Dalum S., Hedegård P. (2019). Theory of
chiral induced spin selectivity. Nano Lett..

[16] Giaconi N., Lupi M., Das T. K., Kumar A., Poggini L., Viglianisi C., Sorace L., Menichetti S., Naaman R., Sessoli R., Mannini M. (2024). Spin Polarized Current
in Chiral Organic Radical Monolayers. J. Mater.
Chem. C.

[17] Naaman R., Paltiel Y., Waldeck D. H. (2019). Chiral
Molecules and the Electron
Spin. Nat. Rev. Chem..

[18] Bloom B. P., Paltiel Y., Naaman R., Waldeck D. H. (2024). Chiral Induced Spin
Selectivity. Chem. Rev..

[19] Zinc Oxide Bulk, Thin Films and Nanostructures; Elsevier, 2006.

[20] Özgür Ü., Alivov Y. I., Liu C., Teke A., Reshchikov M. A., Doğan S., Avrutin V., Cho S.-J., Morkoç H. (2005). A Comprehensive
Review of ZnO Materials and Devices. J. Appl.
Phys..

[21] Klingshirn C. (2007). ZnO: Material,
Physics and Applications. ChemPhysChem..

[22] Ochanda F., Cho K., Andala D., Keane T. C., Atkinson A., Jones W. E. (2009). Synthesis and optical properties of co-doped ZnO submicrometer
tubes from electrospun fiber templates. Langmuir.

[23] Wang Z. L. (2004). Zinc Oxide
Nanostructures: Growth, Properties and Applications. J. Phys.: Condens. Matter.

[24] Rutherford D., Remes Z., Kolarova K., Matolinova I., Cech J., Micova J., Rezek B. (2024). Enhanced Antimicrobial
and Photocatalytic Effects of Plasma-Treated Gallium-Doped Zinc Oxide. Appl. Surf. Sci..

[25] Buryi M., Remeš Z., Babin V., Novotný M., Vaněček V., Aubrechtová Dragounová K., Mičová J., Landová L., Kučerková R., More-Chevalier J., Chertopalov S., Fitl P., Kmječ T. (2021). Influence
of Mo Doping on the Luminescence Properties and Defect States in ZnO
Nanorods. Comparison with ZnO:Mo Thin Films. Appl. Surf. Sci..

[26] Buryi M., Babin V., Remeš Z., Mičová J. (2022). Charge Trapping
Processes in Hydrothermally Grown Er-Doped ZnO. Radiat. Meas..

[27] Sato K., Katayama-Yoshida H. (2002). First Principles Materials Design
for Semiconductor
Spintronics. Semicond. Sci. Technol..

[28] Ronning C., Gao P. X., Ding Y., Wang Z. L., Schwen D. (2004). Manganese-Doped
ZnO Nanobelts for Spintronics. Appl. Phys. Lett..

[29] Pan F., Song C., Liu X. J., Yang Y. C., Zeng F. (2008). Ferromagnetism
and Possible Application in Spintronics of Transition-Metal-Doped
ZnO Films. Mater. Sci. Eng. R Rep..

[30] Chawla A. K., Jain R., Singh J., Mir K. H., Garg T., Rao A. U., Tiwari S. K., Chauhan A., Sardana N., Chawla V., Kumar S. (2022). Sputter Deposited
Mn-doped ZnO Thin
Film for Resistive Memory Applications. ChemistrySelect.

[31] Son D. I., Kwon B. W., Park D. H., Seo W. S., Yi Y., Angadi B., Lee C. L., Choi W. K. (2012). Emissive ZnO–Graphene
Quantum Dots for White-Light-Emitting Diodes. Nat. Nanotechnol..

[32] Schwartz D. A., Norberg N. S., Nguyen Q. P., Parker J. M., Gamelin D. R. (2003). Magnetic
Quantum Dots: Synthesis, Spectroscopy, and Magnetism of Co^2+^ - and Ni^2+^ -Doped ZnO Nanocrystals. J. Am. Chem. Soc..

[33] Duan Y., Han L., Zhang J., Asahina S., Huang Z., Shi L., Wang B., Cao Y., Yao Y., Ma L., Wang C., Dukor R. K., Sun L., Jiang C., Tang Z., Nafie L. A., Che S. (2015). Optically Active Nanostructured
ZnO Films. Angew. Chem., Int. Ed..

[34] Yeom B., Zhang H., Zhang H., Park J. I., Kim K., Govorov A. O., Kotov N. A. (2013). Chiral
Plasmonic Nanostructures on
Achiral Nanopillars. Nano Lett..

[35] Stefanelli M., Magna G., Zurlo F., Caso F. M., Di Bartolomeo E., Antonaroli S., Venanzi M., Paolesse R., Di Natale C., Monti D. (2019). Chiral Selectivity of Porphyrin–ZnO Nanoparticle Conjugates. ACS Appl. Mater. Interfaces.

[36] Ai M., Pan L., Shi C., Huang Z. F., Zhang X., Mi W., Zou J. J. (2023). Spin selection
in atomic-level chiral metal oxide for
photocatalysis. Nat. Commun..

[37] Murakoshi K., Azechi T., Hosokawa H., Wada Y., Yanagida S. (1999). Chiroselective
Electron Transfer at Enantiomer-Capped ZnO Nanocrystalline Surfaces. J. Electroanal. Chem..

[38] Cei M., Di B. L., Zinna F. (2023). Circularly Polarized Luminescence
of Helicenes: A Data-informed Insight. Chirality.

[39] Yang C., Zhu X., Liu M. (2021). Helicenes at air/water interface: spreading film and
metal ion induced a helical ring nanostructure. Langmuir.

[40] Fujikata K., Gon M., Tanaka K., Chujo Y., Tsurusaki A., Kamikawa K. (2023). Synthesis and Properties of Optically Active [7]­Helicene-Fused
Oxanorbornene Polymers by Ring-Opening Metathesis. Macromolecules.

[41] Ernst K. H. (2024). Helicenes
on Surfaces: Stereospecific On-Surface Chemistry, Single Enantiomorphism,
and Electron Spin Selectivity. Chirality.

[42] Stará I. G., Alexandrová Z., Teplý F., Sehnal P., Starý I., Šaman D., Buděšínský M., Cvačka J. (2005). Asymmetric Synthesis of [7]­Helicene-Like Molecules. Org. Lett..

[43] Zhu Y., Xia Z., Cai Z., Yuan Z., Jiang N., Li T., Wang Y., Guo X., Li Z., Ma S., Zhong D., Li Y., Wang J. (2018). Synthesis and Characterization
of Hexapole [7]­Helicene, A Circularly Twisted Chiral Nanographene. J. Am. Chem. Soc..

[44] Safari M. R., Matthes F., Ernst K. H., Bürgler D. E., Schneider C. M. (2022). Deposition of Chiral Heptahelicene Molecules on Ferromagnetic
Co and Fe Thin-Film Substrates. Nanomaterials.

[45] Sahasithiwat S., Mophuang T., Menbangpung L., Kamtonwong S., Sooksimuang T. (2010). 3,12-Dimethoxy-7,8-Dicyano-[5]­Helicene
as a Novel Emissive
Material for Organic Light-Emitting Diode. Synth.
Met..

[46] Hua W., Liu Z., Duan L., Dong G., Qiu Y., Zhang B., Cui D., Tao X., Cheng N., Liu Y. (2015). Deep-Blue Electroluminescence
from Nondoped and Doped Organic Light-Emitting Diodes (OLEDs) Based
on a New Monoaza[6]­Helicene. RSC Adv..

[47] Zhu C., Yang K., Wang H., Fang Y., Feng L., Zhang J., Xiao Z., Wu X., Li Y., Fu Y., Zhang W. (2022). Enantioseparation
in hierarchically porous
assemblies of homochiral cages. ACS Cent. Sci..

[48] Giaconi N., Poggini L., Lupi M., Briganti M., Kumar A., Das T. K., Sorrentino A. L., Viglianisi C., Menichetti S., Naaman R., Sessoli R., Mannini M. (2023). Efficient
Spin-Selective Electron Transport at Low Voltages of Thia-Bridged
Triarylamine Hetero[4]­Helicenes Chemisorbed Monolayer. ACS Nano.

[49] Zhang H. Q., Li J. T., Ma H., Zhang X. B., Zhang G. P., Ren J., Qiu S., Hu G. C. (2026). Pressure-Induced Antiferromagnetic
to Nonmagnetic Phase Transition in a Heptacene Junction. Langmuir.

[50] Izquierdo-García P., Fernandez G. J. M., Medina R. S., Samal M., Rybacek J., Bednarova L., Ramirez B. S., Ramírez F. J., Rodríguez R., Perles J., García F. D. (2023). Helical bilayer nanographenes:
impact of the helicene length on the
structural, electrochemical, photophysical, and chiroptical properties. J. Am. Chem. Soc..

[51] Zhang Y., Lu J., Gao W., Zhang Y., Gao L., Ernst K. H., Cai J. (2026). On-Surface Synthesis of Azapolyarenes and Their Bromine-Assisted
Chiral Self-Assembly. ACS Appl. Mater. Interfaces.

[52] Saito N., Adachi Y., Suzuki T. T. (2026). Crystal
facet engineering and polarity
control in ZnO gas sensors: advancements in sensing performance and
operando surface analysis-secondary publication. Chem. Lett..

[53] Huang M., Weng S., Wang B., Hu J., Fu X., Liu P. (2014). Various Facet Tunable ZnO Crystals
by a Scalable Solvothermal Synthesis
and Their Facet-Dependent Photocatalytic Activities. J. Phys. Chem. C.

[54] Hematian H., Ukraintsev E., Rezek B. (2022). Strong Structural and Electronic
Binding of Bovine Serum Albumin to ZnO via Specific Amino Acid Residues
and Zinc Atoms. ChemPhysChem..

[55] Rezek B., Hematian H., Jíra J., Rutherford D., Kuliček J., Ukraintsev E., Remeš Z. (2021). Microscopic
Study of Bovine Serum Albumin Adsorption on Zinc Oxide (0001) Surface. Phys. Status Solidi A.

[56] Ukraintsev E., Hematian H., Rezek B. (2023). Polarization
Controlled Assembly
of Ultrathin Thiorphan Nanostructures on ZnO Surface Facets. Langmuir.

[57] Jayswal, S. ; Moirangthem, R. S. Thermal Decomposition Route to Synthesize ZnO Nanoparticles for Photocatalytic Application. In AIP Conference Proceedings, Vol. 2009, 2018; p 020023.

[58] Metzger T. S., Batchu H., Kumar A., Fedotov D.vA., Goren N., Bhowmick D. K., Shioukhi I., Yochelis S., Schapiro I., Naaman R., Gidron O., Paltiel Y. (2023). Optical activity and
spin polarization: the surface effect. J. Am.
Chem. Soc..

[59] Xu Y., Duan J. J., Yi Z. Y., Zhang K. X., Chen T., Wang D. (2021). Chirality
of Molecular Nanostructures on Surfaces via Molecular Assembly
and Reaction: Manifestation and Control. Surf.
Sci. Rep..

[60] Houska V., Ukraintsev E., Vacek J., Rybáček J., Bednárová L., Pohl R., Stará I. G., Rezek B., Stary I. (2023). Helicene-Based π-Conjugated
Macrocycles: Their Synthesis, Properties, Chirality and Self-Assembly
into Molecular Stripes on Graphite Surface. Nanoscale.

[61] Ukraintsev E., Houska V., Rezek B. (2022). Small Angle Symmetry Splitting of
Helicene-Based Molecular Wires on Pyrolytic Graphite. Carbon.

[62] Ukraintsev E., Houska V., Vacek J., Starý I., Stará I. G., Rezek B. (2023). Racemic Dimers as Models of Chiral
Macrocycles Self-Assembled on Pyrolytic Graphite. Carbon Trends.

[63] Raula M., Maity D., Rashid M. H., Mandal T. K. (2012). In Situ Formation
of Chiral Core–Shell Nanostructures with Raspberry-like Gold
Cores and Dense Organic Shells Using Catechin and Their Catalytic
Application. J. Mater. Chem..

[64] Ghosh S., Badruddoza A. Z. M., Uddin M. S., Hidajat K. (2011). Adsorption of Chiral
Aromatic Amino Acids onto Carboxymethyl-β-Cyclodextrin Bonded
Fe3O4/SiO2 Core–Shell Nanoparticles. J. Colloid Interface Sci..

[65] Hao C., Wu X., Sun M., Zhang H., Yuan A., Xu L., Xu C., Kuang H. (2019). Chiral Core–Shell Upconversion
Nanoparticle@MOF
Nanoassemblies for Quantification and Bioimaging of Reactive Oxygen
Species in Vivo. J. Am. Chem. Soc..

[66] Carella A., Mishra S., Ferrari C., Vanossi D., Rossella F., Pop F., Avarvari N., Htoon H., Hollingsworth J. A., Bowes E. G., Majumder S., Jones A. C., Fontanesi C. (2025). Chiral Induction
at the Nanoscale and Spin Selectivity in Electron Transmission in
Chiral Methylated BEDT-TTF Derivatives. Nanoscale.

[67] Rezek B., Ukraintsev E., Krátká M., Taylor A., Fendrych F., Mandys V. (2014). Epithelial
Cell Morphology and Adhesion
on Diamond Films Deposited and Chemically Modified by Plasma Processes. Biointerphases.

[68] Nečas D., Klapetek P. (2012). Gwyddion: an open-source
software for SPM data analysis. Central Euro.
J. Phys..

[69] *QuantumATK*, Synopsys QuantumATK version 2024, Synopsys QuantumATK. 2024.

[70] Rappé A. K., Casewit C. J., Colwell K. S., Goddard III W. A., Skiff W. M. (1992). UFF, a full periodic
table force field. J. Am. Chem. Soc..

[71] Senftle T. P., Hong S., Islam M. M., Kylasa S. B., Zheng Y., Shin Y. K., Junkermeier C., Engel-Herbert R., Janik M. J., Aktulga H. M., Verstraelen T. (2016). The ReaxFF reactive force-field: development, applications and future
directions. npj Computational Mater..

[72] Pelaez R. P., Simeon G., Galvelis R., Mirarchi A., Eastman P., Doerr S., Tholke P., Markland T. E., De Fabritiis G. (2024). Torchmd-net
2.0: Fast neural network potentials for molecular simulations. J. Chem. Theory Comput..

[73] Sengul M. Y., Randall C. A., van Duin A. C. T. (2018). ReaxFF Molecular
Dynamics Study on
the Influence of Temperature on Adsorption, Desorption, and Decomposition
at the Acetic Acid/Water/ZnO(101̅0) Interface Enabling Cold
Sintering. ACS Appl. Mater. Interfaces.

[74] Elstner M., Porezag D., Jungnickel G., Elsner J., Haugk M., Frauenheim T., Suhai S., Seifert G. (1998). Self-consistent-charge
density-functional tight-binding method for simulations of complex
materials properties. Phys. Rev. B.

